# Gender disparities among medical students choosing to pursue careers in medical research: a secondary cross-sectional cohort analysis

**DOI:** 10.1186/s12909-021-03004-z

**Published:** 2021-11-25

**Authors:** Austin Snyder, David Xiang, Alison Smith, Shannon Esswein, Omar Toubat, John Di Capua, Jennifer M. Kwan, Dania Daye

**Affiliations:** 1 Harvard Medical School, Boston, USA; 2 Keck School of Medicine of USC, Los Angeles, USA; 3 David Geffen School of Medicine at UCLA, Los Angeles, USA; 4 Department of Interventional Radiology, Massachusetts General Hospital, 55 Fruit Street, Boston, MA 02114 USA; 5 Section of Cardiovascular Medicine, Yale School of Medicine, 333 Cedar Street, New Haven, CT 06510 USA

**Keywords:** Gender disparities, Research, Academic medicine, Work-life balance

## Abstract

**Background:**

Though the proportion of women in medical schools has increased, gender disparities among those who pursue research careers still exists. In this study, we seek to better understand the main factors contributing to the existing gender disparities among medical students choosing to pursue careers in medical research.

**Methods:**

A secondary cross-sectional cohort analysis of previously published data was conducted using a 70-item survey that was sent to 16,418 medical students at 32 academic medical centers, and was IRB exempt from the need for ethical approval at the University of Illinois at Chicago and the University of Pennsylvania. Data was collected from September 2012 to December 2014. Survey results were analyzed using chi-square tests and Cramer’s V to determine gender differences in demographic characteristics (training stage, race/ethnicity, marital status, parental status, financial support, and parental career background), career sector choice, career content choice, specialty choice, foreseeable career obstacles, and perceptions about medical research careers.

**Results:**

Female respondents were more likely to be enrolled in MD-only programs, while male respondents were more likely to be enrolled in MD/PhD programs. More male students selected academia as their first-choice career sector, while more female respondents selected hospitalist as their first-choice career sector. More female respondents identified patient care and opportunities for community service as their top career selection factors, while more male respondents identified research and teaching as their top career selection factors. Student loan burden, future compensation, and work/life balance were the most reported obstacles to pursuing a career in medical research.

**Conclusions:**

There are many factors from a medical student’s perspective that may contribute to the existing gender disparities in pursuing a career in medical research. While much progress has been made in attracting nearly equal numbers of men and women to the field of medicine, active efforts to bridge the gap between men and women in medical research careers are needed.

**Supplementary Information:**

The online version contains supplementary material available at 10.1186/s12909-021-03004-z.

## Introduction

Physician-scientists have long been considered an endangered species and the female physician-scientist an even more rare entity [1]. Despite efforts to attract physicians to medical research, interest has continued to dwindle, especially among female students. Furthermore, women also continue to be underrepresented in leadership and administrative roles in academic medicine [2]. While the causes of these phenomena have long been debated and are certainly multi-factorial, no solutions have been realized.

Furthermore, for reasons that are not well understood, the interest in medical research has been dwindling [3–5]. According to a 2014 report from the NIH, only 1.5% of MDs consider research their primary focus with even fewer physicians receiving funding as principal investigators on NIH grants (0.9%), split evenly between MDs and MD-PhDs [6]. Whereas the number of nonphysician (PhD) NIH-funded investigators has increased by 50% over the last 20 years, the number of NIH-funded physician-scientists has essentially remained constant.

The stagnating physician-scientist workforce has also failed to keep pace with the increase in racial and gender diversity of its MD counterpart. The ever-growing body of evidence continues to support the importance of achieving greater diversity in the biomedical workforce [7]. However, the pipeline remains leaky with striking losses of female talent at higher levels of academic medicine [8, 9]. Historically, the female physician was a rarity. In the 1970s, the proportion of women graduating from US medical schools nearly tripled by the end of that decade [10]. Today, women comprise 46% of residents, yet the proportion of women at the rank of full professor (12%) remains far below that of men [11].

Unfortunately, women are much less likely than their male counterparts to express interest in a career in medical research altogether, at either matriculation or graduation. Furthermore, women who initially express interest in pursuing research as part of their careers are more likely to lose their research career aspirations throughout medical school [12]. The reasons for these disparities are certainly multifactorial and likely include factors such as lack of adequate role models, gender discrimination/bias, and work-life balance, but there remain many inconsistencies in the contribution of these factors to this alarming trend [13]. Although previous studies have identified factors influencing interest in research careers among MD and MD/PhD students [14], this study aims to provide a secondary analysis of this data to investigate gender differences among various factors contributing to medical students’ interest in pursuing medical research.

## Methods

### Data collection

The study was reviewed and IRB exempt from the need for ethical approval at the University of Illinois at Chicago and the University of Pennsylvania. All methods were carried out in accordance with relevant guidelines and regulations. A secondary cross-sectional cohort analysis of previously published data was conducted using a 70-item survey that was designed with feedback from a survey design team at the University of Illinois at Chicago [14, 15]. Data were collected using an online survey tool (SurveyMonkey, www.surveymonkey.com) (Appendix I). The survey tool was piloted at 5 institutions for validation of the survey which lasted 18 months (2011 to 2012) [14]. This data was not included in the present study.

Data collection for the present study was collected from 2012 to 2014. The survey was sent in September 2012 via e-mail to 16,418 MD and MD/PhD students at 32 US academic medical centers through student listservs and the institutional representatives of the American Physician Scientists Association (APSA). There was a phased deployment of the survey during this time with the addition of different institutions each year. Three reminders were sent per institution during the study period.

All identifying information was anonymized by the survey collection tool. Furthermore, the survey collection tool did not allow for more than 1 response from each IP address. To participate in the study, respondents had to be students enrolled in a medical school or graduate school program, or taking a year off for research, which was reflected in the responses to the survey. The denominator of total responses used to calculate the response rate represents the cumulative number of students that the survey was sent out to at the participating institutions. Participants had the option of entering their institutional email address for a chance to receive a $50 Amazon gift certificate. E-mail addresses were kept separate from survey responses to maintain the anonymity of responses.

MD/PhD students were identified through how they paid for medical school as being sponsored by an MD/PhD program. MD candidates interested in research-intensive careers (MD-RI) were identified by their career intentions of wanting a research to clinical duty ratio of 50% or greater, which reflects the NIH guidelines for surgeon scientists. Medical students (years 1–4) were defined as those enrolled in a US medical school. Graduate school students (years 1–5+) were defined as those enrolled in an MD/PhD program. Students taking a year out for research, or a one-year graduate school program were separately categorized by the survey. The primary hypothesis was that there is a difference in barriers to pursuing careers in medical research based on gender. The primary outcome was the various factors contributing to the gender disparities in medical research careers.

### Statistical analysis

Survey results were analyzed to determine significant gender differences in demographic characteristics (training stage, race/ethnicity, marital status, parental status, financial support, and parental career background), career sector choice, career content choice, specialty choice, foreseeable career obstacles, and perceptions about medical research careers. Chi-squared tests were used to measure the significance of associations between categorical variables. Where data did not meet minimum expected cell counts, Fisher’s exact test was performed. Cramer’s V analysis was used to estimate the effect size of statistically significant tests between male and female respondents. The strength of association for Cramer’s V was categorized as follows: ≤0.05 (very weak), > 0.05 (weak), > 0.10 (moderate), > 0.15 (strong), and > 0.25 (very strong) [16]. All tests were performed using SPSS. All tests of significance were 2-sided and *p* < 0.05 was considered significant. Acute care specialties included pulmonary critical care, anesthesiology, and emergency medicine.

## Results

### Demographics

There were 4433 respondents (27% response rate). Demographic characteristics (gender, training stage, race/ethnicity, marital status, parental status, financial support, and parental career background) of respondents are summarized in Table 1.

**Table 1:** Demographics of Female and Male Respondents

Demographic	Female, n (%)	Male, n (%)	***P***-value^**b**^	Cramer’s V^**c**^
Gender Distribution	2328 (56.3%)	1795 (43.4%)		
**Training program**			**< 0.001**	**0.11**
MD-RI	366 (15.7%)	284 (15.8%)		
MD/PhD	394 (16.9%)	459 (25.6%)		
MD Only	1568 (67.4%)	1052 (58.6%)		
TOTAL	2328 (100%)	1795 (100%)		
**Training stage**^**a**^			**0.20**	**N/A**
Medical School Year 1	657 (28.4%)	502 (28.2%)		
Medical School Year 2	576 (24.9%)	462 (26.0%)		
Medical School Year 3	392 (17.0%)	281 (15.8%)		
Medical School Year 4	407 (17.6%)	271 (15.2%)		
Graduate School Year	5 (0.2%)	5 (0.3%)		
Year Out for Research	61 (2.6%)	39 (2.2%)		
Graduate School Year 1	64 (2.8%)	69 (3.9%)		
Graduate School Year 2	49 (2.1%)	54 (3.0%)		
Graduate School Year 3	44 (1.9%)	40 (2.3%)		
Graduate School Year 4	46 (2.0%)	42 (2.4%)		
Graduate School Year 5 or more	11 (0.5%)	14 (0.8%)		
TOTAL	2312 (100%)	1779 (100%)		
**Race**			**0.002**	**0.07**
White	1587 (69.9%)	1263 (72.8%)		
Black or African American	114 (5.0%)	52 (3.0%)		
American Indian or Alaska Native	6 (0.3%)	4 (0.2%)		
Asian or Pacific Islander	259 (11.4%)	159 (9.2%)		
Multi-racial or Other	303 (13.4%)	258 (14.9%)		
TOTAL	2269 (100%)	1736 (100%)		
**Marital status**			**0.07**	**N/A**
Married/Partnered	569 (25.1%)	481 (27.6%)		
Not Married/Partnered	1698 (74.9%)	1261 (72.4%)		
TOTAL	2267 (100%)	1742 (100%)		
**Parental status**			**< 0.0001**	**0.07**
Has a child/children (of 4041)	97 (4.3%)	132 (7.6%)		
Does NOT have a child/children	2168 (95.7%)	1611 (92.4%)		
TOTAL	2265 (100%)	1743 (100%)		
**Primary source of medical school funding**			**< 0.0001**	**0.13**
MD-PhD or DO-PhD sponsored only	345 (15.1%)	403 (22.9%)		
Scholarships	210 (9.2%)	171 (9.7%)		
Grants	37 (1.6%)	36 (2.1%)		
Loans	1238 (54.3%)	874 (49.7%)		
National Service	19 (0.8%)	31 (1.8%)		
Personal Savings	27 (1.2%)	18 (1.0%)		
Family/Partner Support	398 (17.5%)	223 (12.7%)		
Work	2 (0.1%)	4 (0.2%)		
Other	3 (0.1%)	0 (0.0%)		
TOTAL	2279 (100%)	1760 (100%)		

^a^ Excluding Other/NA

^b^ Male versus female responses were compared using chi-squared tests and Fisher’s exact tests where appropriate

^c^ Cramer’s V was used to measure effect size between male and female respondents

#### Gender

Among all respondents, there were more females (2328, 56.3%) than males (1795, 43.4%). Female respondents were more likely to be enrolled MD-only programs (1568, 67.4% versus 1052, 58.6%) while male respondents were more likely to be enrolled in MD/PhD programs (459, 25.6% versus 394, 16.9%). In contrast, an equal proportion of female (366, 15.7%) and male respondents (284, 15.8%) self-identified to be MD-RI as defined by intending a > 50% research/clinical ratio. *P*-value < 0.001 unless otherwise stated (Table 1).

#### Training stage

Survey responses came from students in all stages of MD and MD/PhD programs, including all medical school years, five different graduate school years, and students in a research year program. No significant difference in distribution between males and females within each specific stage of training was observed (*p* = 0.20) (Table 1).

#### Race/ethnicity

The majority of respondents were white (2850, 71.3%). Among male students, significantly more white (1263, 72.8% versus 1587, 70.0%) and multiracial students (258, 14.9% versus 303, 13.4%) responded compared to females. In contrast, among female respondents, more identified as black (114, 5.0% versus 52, 3.0%) or Asian (259, 11.4% versus 159, 9.2%) compared to their male counterparts (*p* = 0.002) (Table 1).

#### Marital status

Most survey respondents were not married/partnered (2959, 73.8%) versus married/partnered students (1050, 26.2%). There were no gender differences between partnered and not partnered students (*p* = 0.07) (Table 1).

#### Parental status

A majority of respondents did not have children (3779, 94.3%) compared to those who had children (229, 5.7%). 132 (7.6%) of male respondents reported having children compared to 97 (4.3%) of female respondents (*p* < 0.0001) (Table 1).

#### Financial support

More males than females paid for their medical training exclusively through program (i.e. MD/PhD or DO/PhD) sponsorships (403, 22.9% versus 345, 15.1%), scholarships (171, 9.7% versus 210, 9.2%), grants (36, 2.1% versus 37, 1.6%), national services (31, 1.8% versus 19, 0.8%), and work (4, 0.2% versus 2, 0.1%). In contrast, more female than male respondents depended upon loans (1238, 54.3% versus 874, 49.7%), personal savings (27, 1.2% versus 18, 1.0%), and family/partner support (398, 17.5% versus 223, 12.7%) (*p* < 0.0001) (Table 1).

### Career intentions

#### Career sector

**Fig. 1:**
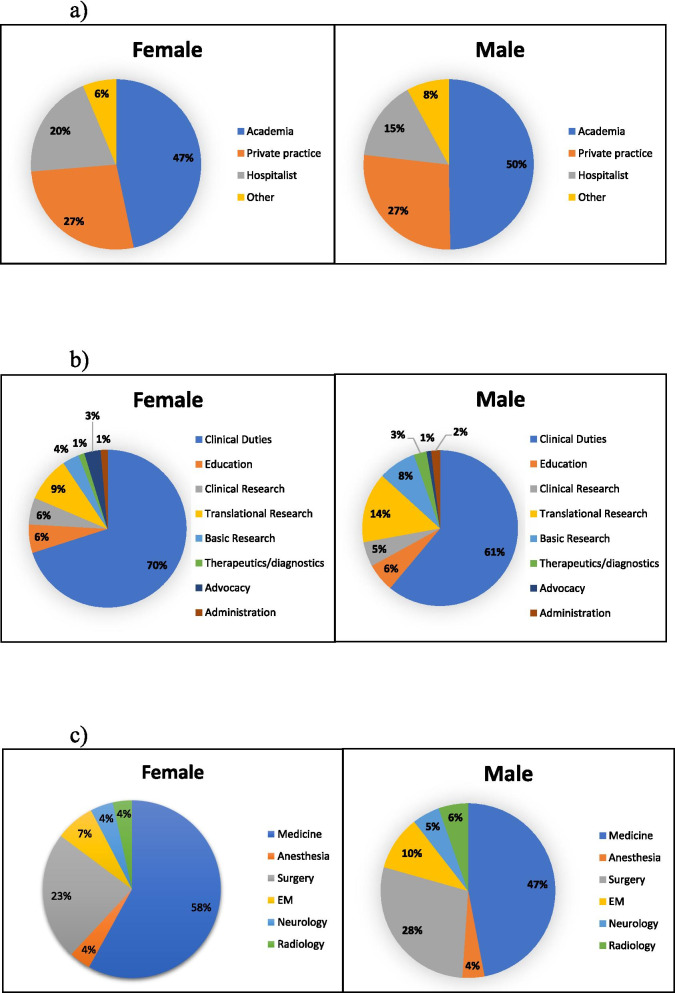
**a** 1st Sector Choice by Gender^**a**^, *P* = 0.0004, Cramer’s V = 0.10. ^**a**^ Top sector choice for participants separated by gender. The following sectors were included in the category “Other” for better visualization: nonprofit, government, industry, and consulting. **b** 1st Career Intention by Gender^**b**^, *P* < 0.0001, Cramer’s V = 0.16. ^**b**^ Top career intention for participants separated by gender. The category “Other/NA” was excluded for better visualization. **c** 1st Specialty of Interest by Gender^**c**^, *P* < 0.0001, Cramer’s V = 0.31. ^**c**^ Top choice specialty of interest for participants separated by gender. The following specialties were included in the category “Medicine” for better visualization: allergy and immunology, dermatology, family medicine, internal medicine, internal medicine subspecialties, medical genetics, pathology, pediatrics, physical medicine and rehabilitation, preventive medicine, and psychiatry. The following specialties were included in the category “Surgery” for better visualization: surgical subspecialties, obstetrics and gynecology, ophthalmology, otolaryngology, and urology. The following specialties were included in the category “Radiology” for better visualization: nuclear medicine and radiation oncology. The category “Other/NA” was excluded for better visualization

More male students selected academia (833, 49.7% versus 1008, 46.7%) as their first-choice career compared to females. In contrast, more female respondents chose hospitalist (432, 20.0% versus 254, 15.2%) careers as their top selection relative to males (*p* = 0.0004) (Fig. 1a).

#### Career content

More females desired clinical duties (1526, 70.1% versus 1013, 61.1%) and advocacy work (73, 3.4% versus 16, 1.0%) as their first career intention compared to male students. Male students, in contrast, chose translational research (242, 14.6% versus 200, 9.2%), basic research (130, 7.8% versus 75, 3.4%), and therapeutics/diagnostics work (44, 2.7% versus 27, 1.2%) as their top career intention compared to females (*p* < 0.0001) (Fig. 1b).

#### Residency specialties: 1st specialty of interest

Significantly more male students preferred surgical specialties (471, 28.3% versus 499, 23.3%), emergency medicine (168, 10.1% versus 153, 7.1%), and radiology (90, 5.4% versus 75, 3.5%) relative to females, while more female respondents chose medical specialties (1245, 58.0% versus 782, 47.0%) as their top intended specialty (*p* < 0.0001) (Fig. 1c).

#### Career selection factors

More male respondents identified research (255, 15.4% versus 169, 7.7%), teaching (60, 3.6% versus 41, 1.9%), financial security (110, 6.6% versus 52, 2.4%) and autonomy (61, 3.7% versus 33, 1.5%) as the top career selection factors. In comparison, more female respondents identified patient care (809, 37.1% versus 510, 30.8%), community service (93, 4.3% versus 29, 1.8%) and work life balance (855, 39.2% versus 539, 32.6%) as the top career selection factors (*p* < 0.0001) (Table 2).

**Table 2:** Top Career Selection Factors by Female and Male Respondents

Factor^**a**^	Female, n (%)	Male, n (%)	***P*** < 0.0001^**b**^	Cramer’s V = 0.21^**c**^
Opportunities to do research	169 (7.7%)	255 (15.4%)		
Opportunities for patient care	809 (37.1%)	510 (30.8%)		
Opportunities to teach	41 (1.9%)	60 (3.6%)		
Opportunities for community service	93 (4.3%)	29 (1.8%)		
Opportunities for interaction with students	20 (0.9%)	16 (1.0%)		
Opportunities for travel	14 (0.6%)	10 (0.6%)		
Opportunities for international work	70 (3.2%)	42 (2.5%)		
Opportunities for national work	8 (0.4%)	8 (0.5%)		
Opportunities for local work	12 (0.6%)	7 (0.4%)		
Ability to balance work and personal life	855 (39.2%)	539 (32.6%)		
Financial security	52 (2.4%)	110 (6.7%)		
Autonomy	33 (1.5%)	61 (3.7%)		
Prestige	7 (0.3%)	7 (0.4%)		
TOTAL	2183 (100%)	1654 (100%)		

^a^ Excluding Other/NA

^b^ Male versus female responses were compared using chi-squared tests and Fisher’s exact tests where appropriate

^c^ Cramer’s V was used to measure effect size between male and female respondents

#### Obstacles

##### Foreseeable work-related obstacles

Though balancing family and work responsibilities was most commonly selected by both males and females as the first foreseeable obstacle, a greater percentage of female respondents (1219, 55.9% versus 709, 42.6%) selected this obstacle. In contrast, a greater percentage of male respondents (202, 12.2% versus 128, 5.9%) identified lack of opportunity/research funding as the top foreseeable obstacle (*p* < 0.0001) (Table 3).

**Table 3:** Obstacles by Female and Male Respondents

**Foreseeable work-related responsibilities after residency**^**a**^	**Female, n (%)**	**Male, n (%)**	***P*** **< 0.0001**^**b**^	**Cramer’s V = 0.18**^**c**^
Lack of opportunity/funding	128 (5.9%)	202 (12.2%)		
Not finding position in desired location	179 (8.2%)	181 (10.9%)		
Loan repayment	319 (14.6%)	210 (12.6%)		
Malpractice/lawsuit	19 (0.9%)	42 (2.5%)		
Under-compensation	65 (3.0%)	74 (4.5%)		
Discrimination/biases against your gender, ethnicity, sexual orientation	34 (1.6%)	12 (0.7%)		
Sexual harassment	2 (0.1%)	0 (0.0%)		
Balancing family and work responsibilities	1219 (55.9%)	709 (42.6%)		
Balancing clinical, research, and education responsibilities	162 (7.4%)	186 (11.2%)		
Satisfactory professional advancement	54 (2.5%)	47 (2.8%)		
TOTAL	2181 (100%)	1663 (100%)		
**Foreseeable non-work-related responsibilities after residency**			***P*-Value**	**Cramer’s V**
Raising children	2048 (88.0%)	1579 (88.0%)	> 0.99	N/A
Taking care of elderly parents	1513 (65.0%)	1150 (64.1%)	0.54	N/A
Being a caretaker to others	657 (28.2%)	595 (33.2%)	0.0007	0.05
Financial support of others	1184 (51.0%)	1017 (56.7%)	0.0002	0.06

^a^ Excluding Other/NA

^b^ Male versus female responses were compared using chi-squared tests and Fisher’s exact tests where appropriate

^c^ Cramer’s V was used to measure effect size between male and female respondents

##### Foreseeable non-work-related responsibilities

More male than female respondents expected to be a caretaker to others (595, 33.2% versus 657, 28.2%, *p* = 0.0007) and financially support others (1017, 56.7% versus 1184, 50.9%, *p* = 0.0002), respectively (Table 3).

### Perceptions

#### Intended research/clinical work ratio

Female students preferred to have no research component (558, 24.3% versus 348, 19.8%) or 25%-time commitment (1047, 44.6% versus 747, 42.4%), while male students preferred 50% research commitment (309, 17.6% versus 370, 16.1%), 75% research commitment (319, 18.1% versus 291, 12.7%) or full-time research (38, 2.2% versus 29, 1.3%) (*p* = 0.03) (Table 4).

**Table 4:** Perceptions of Research/Clinical Work Ratio, Feasibility, and Mentoring

**RI Ratio (Research/Clinical Work)**^**a**^	**Female, n (%)**	**Male, n (%)**	***P*** **= 0.03**^**b**^	**Cramer’s V = 0.10**^**c**^
0%	558 (24.3%)	348 (19.8%)		
25%	1047 (44.6%)	747 (42.4%)		
50%	370 (16.1%)	309 (17.6%)		
75%	291 (12.7%)	319 (18.1%)		
100%	29 (1.3%)	38 (2.2%)		
TOTAL	2295 (100%)	1761 (100%)		
**How feasible is a research intense career in acute care medicine specialties?**			***P*** **< 0.0001**	**0.08**
Highly feasible	130 (5.9%)	118 (6.9%)		
Feasible	750 (33.9%)	494 (29.0%)		
Difficult	945 (42.7%)	700 (41.0%)		
Highly difficult	359 (16.2%)	359 (21.1%)		
Impossible	30 (1.4%)	33 (1.9%)		
TOTAL	2214 (100%)	1704 (100%)		
**How feasible is a research intense career in surgical specialties?**			***P*** **< 0.0001**	**0.10**
Highly feasible	156 (7.1%)	98 (5.7%)		
Feasible	707 (32.0%)	466 (27.3%)		
Difficult	799 (36.1%)	588 (34.5%)		
Highly difficult	494 (22.3%)	471 (27.6%)		
Impossible	56 (2.5%)	83 (4.9%)		
TOTAL	2212 (100%)	1706 (100%)		
**How much importance is given to talents/accomplishments when recruiting applicants for jobs and/or positions in science and medicine?**			***P*** **= 0.30**	**N/A**
A great deal of importance	669 (30.7%)	519 (31.1%)		
A lot of importance	1070 (49.1%)	789 (47.2%)		
Moderate amount of importance	410 (18.8%)	327 (19.6%)		
Little importance	28 (1.3%)	35 (2.1%)		
None at all	1 (0.1%)	1 (0.1%)		
TOTAL	2178 (100%)	1671 (100%)		
**How much importance is given to connections/networking when recruiting applicants for jobs and/or positions in science and medicine?**			***P*** **= 0.01**	**0.06**
A great deal of importance	721 (33.0%)	527 (31.5%)		
A lot of importance	946 (43.4%)	675 (40.4%)		
Moderate amount of importance	456 (20.9%)	406 (24.3%)		
Little importance	59 (2.7%)	62 (3.7%)		
None at all	0 (0.0%)	2 (0.1%)		
TOTAL	2182 (100%)	1672 (100%)		

^a^ Excluding Other/NA

^b^ Male versus female responses were compared using chi-squared tests and Fisher’s exact tests where appropriate

^c^ Cramer’s V was used to measure effect size between male and female respondents

#### Feasibility of research in acute care and surgical specialties

More female than male respondents (750, 33.9% versus 494, 29.0%) believe that research intensive careers in acute care specialties are feasible, while more male than female respondents (359, 21.1% versus 359, 16.2%) believe that research intensive careers in acute care are highly difficult (*p* < 0.0001). As for surgical specialties, more females than males perceive research intensive careers as highly feasible (156, 7.1% versus 98, 5.7%) or feasible (707, 32.0% versus 466, 27.3%), while more males than females believe research intensive careers in surgical specialties are highly difficult (471, 27.6% versus 494, 22.3%), or impossible (83, 4.9% versus 56, 2.5%) (*p* < 0.0001) (Table 4).

#### Perceived important factors in job recruitment

More female than male respondents perceived connections/networking to be “a great deal of importance” (721, 33.0% versus 527, 31.5%), whereas more males than females perceived connections/networking to be of “moderate amount of importance” (406, 24.3% versus 456, 20.9%) (*p* = 0.01) (Table 4).

## Discussion

There are significant gender disparities in many factors surrounding medical student interest in research. Notably, more males were found to pay for medical school through MD/PhD or DO/PhD program funding, scholarships, grants, and national service, thus leading to a significantly reduced financial/loan burden. The study also found that more females identify loan repayment as a top foreseeable obstacle to pursuing medical research compared to males, consistent with current literature which states that female matriculation rates consistently remain below 50% among all MSTP programs [17]. Our data further support this as male respondents were more likely to be enrolled in MD/PhD programs (25.6% vs 16.9%) compared to female respondents.

Furthermore, fewer females identified under-compensation as a top foreseeable obstacle to pursuing a career in research. However, females in the medical profession continue to experience the wage gap which persists through all sectors of society [18]. There may be many reasons for this discordance, one of which may be societal pressure on women to fulfill the heteronormative gender role as the primary caregiver for the family and children, though the contribution of this factor is still debated [13]. Our study further supports this possibility in the finding that a greater percentage of females chose “balancing family and work responsibilities” as the number one factor for both their specialty choice and foreseeable obstacle in pursuing research.

Another notable finding in our study was that while more females saw research intensive careers in surgical and acute care specialties as feasible, fewer females indicated an intent to pursue basic and translational research. This discrepancy is consistent with the continued minority of women entering surgical and acute care specialties, despite recent parity in absolute numbers of students entering the resident workforce [19].

In conclusion, there are a multitude of factors that contribute to the continued disparities in interest in pursuing a career in medical research among medical students. Although several gender differences were observed, the majority of respondents reported a desire to pursue academic medicine, clinical duties, and medical specialties, along with future expectations to raise children and take care of elderly parents. Notable factors that significantly differed by gender included financial burden from student loans, under-compensation, and work/life balance among other factors. Without concerted efforts to bridge this gap between men and women, these disparities will persist. With the current focus on inclusion and diversification in academic medicine, these efforts must target minority populations to ensure these changes come to fruition in the coming years [20, 21].

## Limitations

Although this is a large secondary cross-sectional cohort analysis of previously published data, with a total of 4433 respondents from a nationally representative cohort of medical schools, there are a few limitations to this study. First, given the nature of self-reported surveys, there is the inherent limitation of being unable to assess more deeply the motivations behind who chose to respond to the survey, along with the answers of the respondents. Second, with a response rate of 27%, there are limitations to the generalizability of the study results. Third, we did not impute incomplete responses or responses labeled “N/A” to better reflect the answers of all respondents, which we recognize as a statistical limitation of the study. Fourth, many steps have been taken to promote gender equity in recent years, and given that the survey data was collected between 2012 and 2014, the factors considered by students may have changed since that time. However, we believe many of the obstacles discussed in this study are likely still relevant and timely. Fifth, there are inherent limitations to the study given that this was a secondary analysis of previously published data using a survey that was designed to assess factors influencing student interest in research careers, rather than specifically looking at gender differences in these various factors. Finally, a follow-up study investigating the ultimate career choice of respondents would be helpful to gain perspective on how to best address gaps in those pursuing careers in medical research.

## Supplementary Information

**Additional file 1.**

## Data Availability

The datasets used and/or analyzed during the current study are available from the corresponding authors on reasonable request.

## Abbreviation

APSA: American Physician Scientists Association
